# Novel tool to quantify with single-cell resolution the number of incoming AAV genomes co-expressed in the mouse nervous system

**DOI:** 10.1038/s41434-021-00272-8

**Published:** 2021-06-28

**Authors:** Carola J. Maturana, Jessica L. Verpeut, Mahdi Kooshkbaghi, Esteban A. Engel

**Affiliations:** 1 Princeton Neuroscience Institute, Princeton University, Princeton, NJ USA; 2 Simons Center for Quantitative Biology, Cold Spring Harbor Laboratory, Cold Spring Harbor, NY USA

**Keywords:** Virology, Genetic vectors

## Abstract

Adeno-associated viral (AAV) vectors are an established and safe gene delivery tool to target the nervous system. However, the payload capacity of <4.9 kb limits the transfer of large or multiple genes. Oversized payloads could be delivered by fragmenting the transgenes into separate AAV capsids that are then mixed. This strategy could increase the AAV cargo capacity to treat monogenic, polygenic diseases and comorbidities only if controlled co-expression of multiple AAV capsids is achieved on each transduced cell. We developed a tool to quantify the number of incoming AAV genomes that are co-expressed in the nervous system with single-cell resolution. By using an isogenic mix of three AAVs each expressing single fluorescent reporters, we determined that expression of much greater than 31 AAV genomes per neuron in vitro and 20 genomes per neuron in vivo is obtained across different brain regions including anterior cingulate, prefrontal, somatomotor and somatosensory cortex areas, and cerebellar lobule VI. Our results demonstrate that multiple AAV vectors containing different transgenes or transgene fragments, can efficiently co-express in the same neuron. This tool can be used to design and improve AAV-based interrogation of neuronal circuits, map brain connectivity, and treat genetic diseases affecting the nervous system.

## Introduction

AAV-based gene therapies are well established to treat inherited and acquired diseases with known nonfunctional or dysfunctional gene products [1]. In addition, AAV vectors are essential tools in neuroscience research to identify and manipulate neuronal circuits in animal models. AAV vectors are typically used to trace cell circuits, measure neuronal dynamics such as intracellular levels of calcium (GCaMP), glutamate (iGluSnFR), γ-aminobutyric acid (iGABASnFR) and dopamine (dLight) among other applications. Furthermore, precise, conditional spatiotemporal nerve circuit manipulation leading to the understanding of complex biological behaviors is now possible with in vivo AAV-based delivery of chemogenetic (DREADD) and optogenetic (channelrhodopsins) tools [2, 3]. Three AAV-based gene therapies are already approved, and a significant number are in phase III clinical trial [1, 4, 5]. Recombinant AAV vectors provide efficient gene transfer, broad serotype-dependent tropism, low risk of insertional mutagenesis, and long-term transgene expression in transduced cells [1]. However, the packaging capacity (<4.9 Kb) hinders the delivery of large payloads for neuroscience research and the treatment of diseases caused by mutations in large or multiple genes [5, 6]. Therefore, AAV vectors are not ideal to treat (i) disorders caused by mutations in single-oversized genes such as Duchenne muscular dystrophy, retinopathies and deafness [7–9]; (ii) non-monogenic disorders such as Parkinson’s disease, multiple sclerosis and cancer, and non-genetic causes such as HIV/AIDS [4, 10]; and (iii) comorbidities such as age-related diseases (obesity, diabetes, kidney and heart failure) [11]. Different strategies have been developed to deliver large transgenes with AAVs such as the use of small gene promoters and regulatory elements [12]. An alternative approach to deliver oversized genes consists of splitting the transgenes into multiple smaller fragments that are packaged into separate AAV capsids [6, 7]. These fragments combined could potentially reconstitute the full-length gene in the target cell by trans-splicing or homologous recombination between overlapping sequences [6]. Co-administration of AAV mixtures has been assessed to treat non-monogenic diseases and comorbidities affecting brain, spinal cord, heart and kidney [10, 11, 13]. Transgene expression relies on the number of vector genome-containing particles (VGP) required for transduction, a factor that can vary between 25 and several hundred for different cell-types and AAV serotypes [14]. VGP is affected by rate-limiting factors including capsid entry, endosomal escape, nuclear transport, capsid uncoating and second-strand synthesis of single-stranded genomes [14, 15]. Self-complementary AAV (scAAV) vectors avoid the second strand synthesis bottleneck, but further reduce in half the already limited packaging capacity. Kobiler and collaborators developed a method to determine the number of incoming alphaherpesvirus genomes able to express in infected cells in vitro [16]. The authors used a mixture of three fluorescent pseudorabies virus (PRV) recombinants and assumed that viral gene expression is best represented as a Poisson probability. They determined that the maximal average number of PRV genomes expressed in each infected cell (Poisson random variable defined as Lambda (λ)), was approximately 7, thus confirming the presence of multiple bottlenecks during PRV infection [16]. Similarly, studies on human immunodeficiency virus (HIV), showed a multiplicity of infection (MOI) of 3-4 viral copies per cell during coinfection by cell-free virus that like PRV, followed a random Poisson distribution [17]. Moreover, it has been shown that high MOI with HIV can lead to massive recombination events that help to recover from deleterious viral mutations and relentless bottlenecks while simultaneously increasing immune recognition by infected cells [18]. In line with these studies in PRV and HIV, we aimed to determine λ for AAV in neurons to facilitate functional studies of the nervous system and increase the success rate of AAV-based gene therapies.

## Results and discussion

**Fig. 1:**
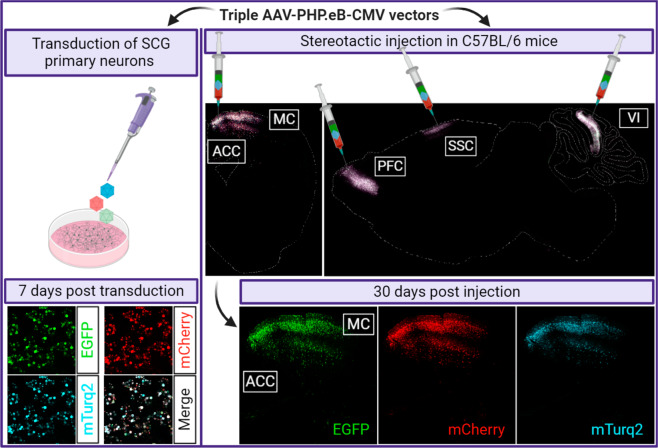
Co-transduction with a fluorescent AAV mixture to estimate the number of incoming genomes co-expressed in neurons in vitro and in vivo. SCG primary neuronal cultures in vitro and mouse brain tissue in vivo were co-transduced with triple AAV mixtures either directly (SCG) or by stereotactic injection in anterior cingulate cortex (ACC), somatomotor cortex (MC), prefrontal cortex (PFC), somatosensory cortex (SSC) and cerebellar lobule VI (VI). Representative confocal images show EGFP, mCherry and mTurquoise2 (mTurq2) fluorescence in SCG neurons at 7 days post AAV transduction (5 × 10^11^ vg/dish), and coronal brain sections at 30 days post AAV injections (400 nl per each brain region, 1.2 x 10^13^ vg/ml). Figure created with BioRender.com.

Here, we quantified with single-cell resolution, the number of incoming viral genomes expressed in neurons in vitro and in vivo after co-transduction with three isogenic AAV vectors, each expressing different fluorescent proteins. The AAV mix was used to probe the number of cells expressing three, two, one, or zero fluorescent reporter. AAV-PHP.eB, an engineered capsid variant, derived from serotype 2/9, was selected for this study due to the pan-neuronal tropism [12, 19]. Primary superior cervical ganglia (SCG) neuronal cultures were transduced with an equimolar mixture of AAV-EGFP, AAV-mCherry and AAV-mTurquoise2 (mTurq2) (1:1:1 ratio) at doses ranging from 1 × 10^10^ to 5 × 10^11^ vector genomes per dish (vg/dish) for 7 days (*n* = 3 per viral dose) (Fig. 1). The same vector mix was then stereotaxically injected into five different brain areas of adult mice including anterior cingulate, prefrontal, somatomotor and somatosensory cortex, and cerebellar lobule VI. Mice were sacrificed at 30 days post injection (dpi) and EGFP, mCherry, mTurq2 labeled cells were clearly identified around the injection sites (Fig. 1).

**Fig. 2:**
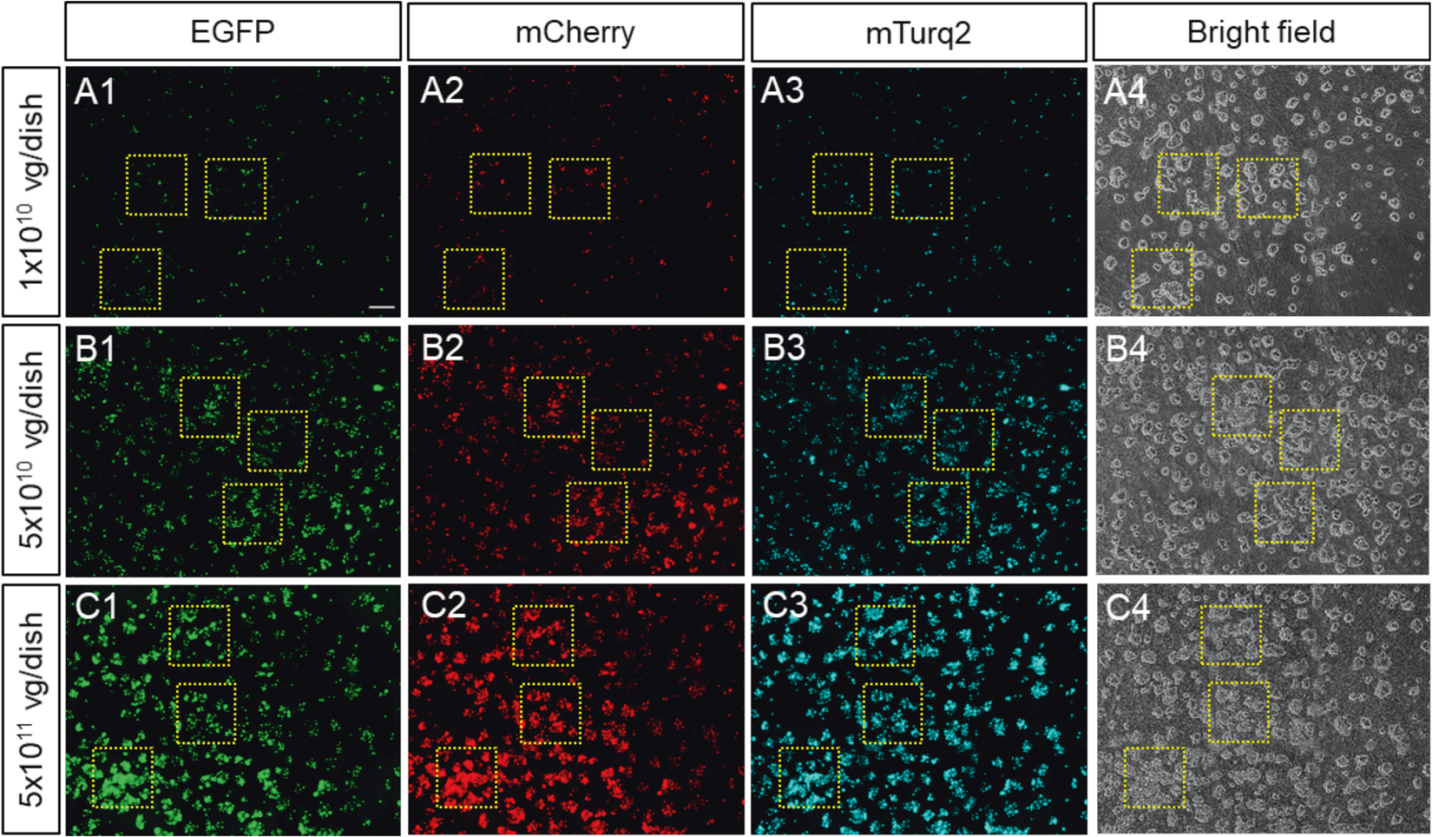
Co-transduction of primary SCG neurons with a fluorescent AAV mixture. SCG neurons were co-transduced with 1 × 10^10^, 5 × 10^10^ and 5 × 10^11^ AAV vg/dish. Fluorescent reporter expression was measured in three regions per cultured dish (yellow squares) for: (A1, B1, C1) EGFP, (A2, B2, C2) mCherry, (A3, B3, C3) mTurq2, and (A4, B4, C4) bright field respectively (*n* = 3). Scale bar, 100 µm.

**Fig. 3:**
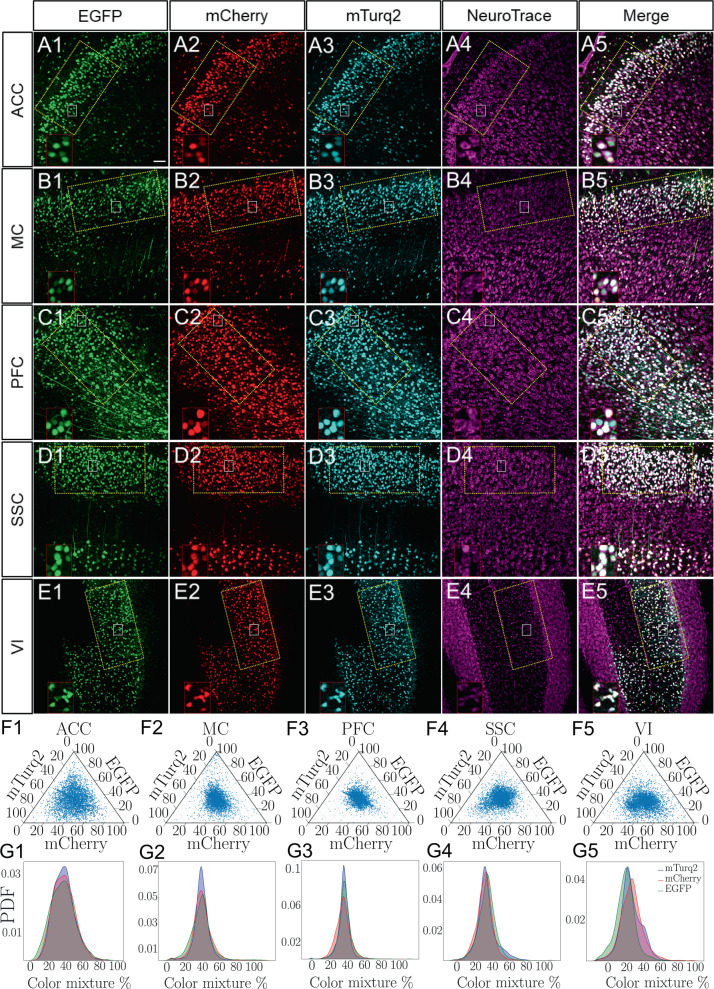
Mouse brain co-transduction with AAV mixtures. **A**–**E** Fluorescent reporter profile of neurons in different brain areas: (A1, B1, C1, D1, E1) EGFP, (A2, B2, C2, D2, E2) mCherry and (A3, B3, C3, D3, E3) mTurq2. (A 1-5) ACC, (B 1-5) MC, (C 1-5) PFC, (D 1-5) SSC and (E 1-5) VI. (A4, B4, C4, D4, E4) Depict labeling with the pan-neuronal marker NeuroTrace. (A5, B5, C5, D5, E5) Show merge of all channels (white signal). Yellow squares depict regions considered for post-processing (*n* = 3). Scale bar, 50 μm. Smaller white squares, show higher magnification insets. (**F** 1–5) Show ternary plots containing the color spectrum of >3900 cells (blue dots) for each brain area. Cells with one- and two-colors are represented by blue dots at the vertices and edges respectively while cells containing mixtures of all three fluorescent reporters are blue dots located inside the triangle. (**G** 1–5) Quantitative representation of the cell color distribution is depicted as probability density functions (PDF) for each brain region shown in F1–F5 respectively.

At both, the 5 × 10^10^ and the 5 × 10^11^ AAV vg/dish doses, all the SCG neurons in the dish showed intense fluorescent reporter expression. That was not the case for the lower dose of 1 × 10^10^ vg/dish. We counted the number of cells expressing three, two, one, or zero fluorescent reporters with single-cell resolution. Notably, the expression of all three reporter genes colocalized in a significant proportion of the transduced neurons in a dose-dependent fashion (yellow squares, *n* = 3 per area) (Fig. 2A–C). Single or double-labeled cells corresponded to less than 10% of the cells quantified, whereas no fluorescent label (zero positive) was detected in less than 1% of the neurons for all viral doses (Table 1). The number of neurons expressing all three reporters reached 100% at 5 × 10^11^ vg/dish and no cells with zero-, one- and two-colors were observed (Table 1). Therefore, the λ value at the highest viral dose was singular (λ → ∞). To obtain a lower bound on λ, we used simple inference for cells with two-colors based on the measurements for lower viral doses. The extrapolation of the two-color counts estimated that λ was much greater than 31 (λ ≫ 31) (Table 1). These results further demonstrate that despite the bottlenecks affecting transgene expression, very high transduction efficiency can be achieved. Next, we performed stereotactic AAV brain injections to assess the in vivo AAV co-transduction efficiency. As shown in Fig. 3, significant neuronal co-expression of all three reporters was observed in every brain area tested (*n* = 3). The vast majority of transduced cells corresponded to neurons that co-labeled with a pan-neuronal marker (Fig. 3 A4, B4, C4, D4, E4). To determine the transduction efficiency for each brain area, the relative fluorescence intensity of more than 3900 neurons across six tissue sections was obtained (Table 2). Each blue dot depicted in the ternary plots, correspond to the percentage values for each reporter gene in which the fluorescent intensity can be found by projecting the dot to the respective triangle edge (axis) (Fig. 3F1-5). We observed equal distribution for all three colors, scattering around the center of the ternary plot. Only a small proportion of cells showed single or double reporter signal. Moreover, we depicted the probability density functions (PDFs), which further confirmed that areas near the brain injection site contained all three fluorescent reporters in equal proportion, each peaking at ~33% in the color mixture percentage (Fig. 3G1-G5). As shown in Table 2, the percentage of brain neurons expressing one or two colors was less than 1%, while neurons with zero color were not observed. Over 99% of the neurons analyzed, showed expression of all three reporters, with an average λ value of ~20 for all brain areas (Table 2). Our findings confirm that a single viral dose containing a mixture of AAV capsids can achieve high co-transduction efficiency both in vitro and in vivo. Consequently, it is safe to assume that AAV vectors can efficiently and simultaneously co-deliver three or more transgenes to study and manipulate the nervous system or treat diseases. Thus, we provide a tool to precisely determine the degree of AAV transduction and transgene expression with single-cell resolution in the nervous system. This tool can be used for the design and optimization of AAV-based gene therapies. Future studies may build on the quantitative data presented here to expand both basic research and therapeutic applications with AAV vectors not only in the brain but also in other organs to deliver either multiple or very large transgenes.

**Table 1:** Estimation of the maximal number of incoming AAV genomes expressed in transduced neurons in vitro (λ).

Infectious dose (vg/dish)	Zero-color (%)	One-color (%)	Two-color (%)	Three-color (%)	Total	λ
1 × 10^10^	14 (0.80 %)	77 (4.40 %)	184 (10.51 %)	1475 (84.29 %)	1750	7.9
5 × 10^10^	5 (0.28 %)	9 (0.51%)	52 (2.93 %)	1707 (96.28 %)	1773	12.4
5 × 10^11^	0 (0.00 %)	0 (0.00 %)	0 (0.00 %)	1791 (100 %)	1791	≫ 31

**Table 2:** Estimation of the maximal number of incoming AAV genomes expressed in transduced neurons in vivo (λ).

Brain area	Zero-color (%)	One-color (%)	Two-color (%)	Three-color (%)	Total	λ
Anterior cingulate cortex	0 (0.00%)	0 (0.00%)	20 (0.50%)	3948 (99.50%)	3968	19.2
Somatomotor cortex	0 (0.00%)	7 (0.16%)	12 (0.28%)	4319 (99.56%)	4338	18.6
Prefrontal cortex	0 (0.00%)	3 (0.07%)	13 (0.29%)	4443 (99.64%)	4459	19.7
Somatosensory cortex	0 (0.00%)	5 (0.1%)	7 (0.15%)	4712 (99.75%)	4724	20.2
Cerebellar lobule VI	0 (0.00%)	4 (0.09%)	15 (0.35%)	4397 (99.56%)	4316	19.0

## Materials and methods

### Construction of AAV Vectors

AAV plasmids containing EGFP, mCherry and mTurq2, driven by the CMV promoter and terminated with SV40 polyA signal were produced by the PNI Viral Core Facility (Princeton Neuroscience Institute, Princeton University). AAV plasmids were produced by either restriction-enzyme cloning, or Gibson assembly. DNA synthesis was provided by GenScript (Piscataway, NJ, USA). AAV plasmids were packaged into AAV serotype PHP.eB as previously described [12, 19]. Fluorescent proteins were fused to the 27 bp triple tandem nuclear localization signal (NLS) derived from human c-myc. All three AAV plasmids generated in this study are publicly available on www.addgene.org under accession numbers 165441, 165442, and 165443 respectively.

### Animals

Pregnant Sprague-Dawley rats (*n* = 3) (Hilltop Labs Animals, Scottdale, PA, USA), were used for SCG isolation and culture in vitro. Four-week-old male C57BL/6 J mice (*n* = 4) were obtained from The Jackson Laboratory (Bar Harbor, ME, USA) for the in vivo experiments. All mice were raised on a 12-h light/dark cycle (lights on at 7:00 am) with *ad libitum* food and water. Special care was taken to minimize suffering and to reduce the number of animals used to the minimum required for statistical inference. Protocols were approved by the Princeton University Institutional Animal Care and Use Committee (protocols 1943-19 and 1947-19).

### SCG neuron culture and AAV transduction

SCG neuronal cultures were obtained from rat embryos as previously described [12]. SCG neurons were cultured for 10 days prior to triple AAV transduction with an equimolar mixture of 1 × 10^10^, 5 × 10^10^ and 5 × 10^11^ vg/dish. Seven days post transduction, neurons were fixed and imaged in a Nikon Ti-E inverted epifluorescence microscope (Nikon Instruments, Tokyo, Japan), with a CoolSNAP ES2 camera (Photometrics, Tucson, AZ, USA) and the Nikon NIS-Elements software.

### Mouse surgery and AAV injection

Mice were anesthetized with isoflurane inhalation (5% isoflurane in O_2_ for induction and 1-2% for maintenance) and placed in a stereotaxic apparatus (Kopf Model 1900, David Kopf Instruments, Tujunga, CA, USA). For stereotactic intracranial brain injections, a midline incision was made followed by craniotomies with a 0.5 mm micro-drill burr (Fine Science Tools, North Vancouver, Canada). Bilateral injections containing 400 nl of the AAV mixture per brain region (1.2 x 10^13^ vg/ml) were made in coordinates (mm), PFC- AP: + 1.8, ML: 0.0, DV: − 2.5; ACC- AP: + 0.8, ML: + 0.35, DV: − 1.5; SSC- AP: − 0.9, ML: − 2.0, DV: − 1.5 and unilateral injection in Lobule VI- AP: − 6.96, ML: 0.0, DV: −0.5, with borosilicate glass capillaries with an outer diameter of 1 mm and an internal diameter of 0.58 mm (World Precision Instruments, Sarasota, FL, USA). These were pilled using the Sutter Micropipette Puller (Model P-2000, Sutter Instrument Company, Novato, CA, USA) and beveled at a 45-degree angle. Craniotomies and skin were sutured, and animals were sacrificed 30 days post injection (dpi) with ketamine (400 mg/kg)/xylazine (50 mg/kg) followed by intraperitoneal 4% PFA perfusion.

### Brain processing

Brains were post-fixed with 4% PFA for 2 h at RT and infiltrated with increasing concentrations of sucrose as previously described [12]. Brains were divided into two parts, right hemispheres were coronally sectioned and left hemispheres were sagittally sectioned at 50 µm using a Leica VT1200 vibratome (Leica Microsystems, Wetzlar, Germany). Brain free-floating sections were permeabilized with 0.5 % Triton X-100 in PBS and incubated in 1:300 NeuroTrace 640/660 deep-red fluorescent Nissl stain (Molecular Probes, Eugene, OR, USA), in PBS containing 0.5 % Triton X-100 for 4 h. Nuclei were counter-stained with 0.5 mg/mL DAPI (Thermo Fisher Scientific, Rockford, IL, USA) and mounted in Vectashield Vibrance antifade mounting media (Vector Laboratories, Burlingame, CA, USA). Sections were analyzed with a Leica SP8-LSCM confocal microscope (Leica Microsystems, Wetzlar, Germany) with a 20X objective and 0.5 μm z steps. Z-stacks were generated with the ImageJ software [20].

### Image analysis

To measure the number of AAV genomes expressed in neurons in vitro, at least 1,750 SCG cells obtained from three replicate dishes per viral dose, were analyzed. Three random image areas were selected for each SCG cultured dish and approximately 200 cells were analyzed for each area. For the in vivo brain experiments, 500 pixel^2^ regions within each AAV-transduced area were selected. At least 250 cells were analyzed for each of the six tissue sections collected per animal for a total of more than 3,900 cells analyzed per brain region and a total of 21,805 cells analyzed for all brain regions. Cells were selected by drawing a region of interest (ROI) in the bright field or NeuroTrace channel and normalized to the background intensity of non-fluorescent cells as previously described [16]. Briefly, the relative fluorescence intensity of each ROI was determined with the following formula: corrected total cell fluorescence = integrated density - ([area of selected cell] x [mean fluorescence of background readings]) [21].

Assuming a Poisson distribution, we estimated the average number of expressed genomes in each cell as:

$$\lambda = - 3\ln \left( {1 - \frac{{r_1 + 2r_2 + 3r_2}}{{3\left( {r_0 + r_1 + r_2 + r_3} \right)}}} \right)$$

where, *r*_0_, *r*_1_, *r*_2_ and *r*_3_ correspond to zero-, single-, double- and triple-colored cells respectively [16]. For post-processing and visualization analyzes, Python 3.7 standard libraries and python-ternary were used [22].
